# Transcriptome analysis revealed the possible regulatory pathways initiating female geese broodiness within the hypothalamic-pituitary-gonadal axis

**DOI:** 10.1371/journal.pone.0191213

**Published:** 2018-02-06

**Authors:** Hehe Liu, Jiwen Wang, Liang Li, Chunchun Han, Hua He, Hengyong Xu

**Affiliations:** Farm Animal Genetic Resources Exploration and Innovation Key Laboratory of Sichuan Province, Sichuan Agricultural University, Chengdu, Sichuan, P.R. China; University of Illinois, UNITED STATES

## Abstract

Geese have the strongest tendency toward broodiness among all poultry. The mechanisms initiating broodiness within the goose hypothalamic-pituitary-gonadal axis (HPGA) are still unclear. Here, we reported the transcriptome differences between laying and initial nesting within the HPGA tissues of geese. We constructed a unigene database based on HPGA tissues and identified 128,148 unigenes, 100% of which have been annotated. By using Digital Gene Expression (DGE) sequencing, we screened 19, 110, 289, and 211 differentially expressed genes (DEGs) in the hypothalamus, pituitary gland, stroma ovarii, and follicles, respectively, between laying and nesting geese. Expression changes of hypocretin (HCRT) and pro-opiomelanocortin (POMC) in the hypothalamus of nesting geese may cause appetite reduction, which is possibly the first step and a prerequisite to initiate broodiness. In addition to prolactin (PRL), follicle-stimulating hormone (FSH) and luteinizing hormone (LH), genes including oxytocin-neurophysin (OXT), chordin-like protein 1 (CHRDL1) and growth hormone (GH), expressed in the pituitary gland, are new candidate molecules that may be involved in broodiness in geese. Heme oxygenase 1 (HMOX1) in the pituitary gland, the proto-oncogene c-Fos (FOS), heat shock protein 90-alpha (HSP90AA), and cyclin-dependent kinase 1 (CDK1) in the ovary that may consolidate and transduce signals regulating the HPGA during broodiness in geese.

## Introduction

Geese *(Anas cygnoides*) are reared commercially as farm birds in the southern parts of China. However, poor laying performance is hindering the progress of the goose industry. As seasonally reproducing waterfowl, geese have the strongest tendency toward broodiness among all poultry, which directly causes a drop in egg production and reproductive efficiency.

The hypothalamic-pituitary-gonadal axis (HPGA) is a coordinated neuroendocrine system that regulates the initiation, development and maintenance of the broodiness process in poultry [1]. In detail, the hypothalamus is the coordinating center of the endocrine system, which consolidates signals derived from upper cortical inputs, autonomic functions, environmental cues such as light and temperature [2], and peripheral endocrine feedback [3]. In turn, the hypothalamus delivers precise signals to the pituitary gland, which then releases hormones that influence most endocrine systems in the body, e.g., the hypothalamus can secrete gonadotropin-releasing hormone (GnRH) to modulate the release of luteinizing hormone (LH) and follicle-stimulating hormone (FSH) in the pituitary gland, thereby controlling gonadal development and sex hormones [4, 5]. In addition, some other hormones secreted from the hypothalamus, including gonadotropin-inhibitory hormone (GnIH), vasoactive intestinal peptide (VIP), and neuropeptide Y (NPY), can also shape gonadal development and the production of sex hormones by modulating pituitary hormone secretion [6].

In recent years, many genes that may have potential roles in regulating broodiness in geese have been identified in HPGA tissues. Luan et al. (2014) screened the differentially expressed genes in the hypothalamus by suppression subtractive hybridization (SSH) between the laying and nesting period and identified GnRH-related genes, such as AdipoR2, Nrg1 and NCAM1, that may be involved in the regulation of nesting in geese [7]. Using next-generation sequencing, Chen et al. (2014) identified the differentially expressed miRNAs in the hypothalamic tissues and found 114 and 94 novel miRNAs in the broody and egg-laying groups, respectively. Moreover, among these, 4 novel miRNAs were differentially expressed between the two groups [8]. Gao et al. (2015) analyzed the transcriptome in the hypothalamus and concluded that the transcripts of a serine/threonine protein kinase (AMPK), heat shock protein 70 (HSP70) and NADH dehydrogenase 1 (ND1) were differentially expressed during the pre-laying and laying periods of geese [9]. Yen et al. (2006) compared the differentially expressed genes in the pituitary gland between pre-laying and laying geese and found that at least 19 genes, as well as prolactin and visinin-like protein, were highly expressed in laying geese compared with pre-laying geese [10]. In the ovarian tissues, Luan et al. (2014) enriched the key regulatory genes between the ceased and laying periods and identified 344 and 344 up- and down-regulated genes, respectively [11]. These studies have compared gene expression between laying and nesting geese and have confirmed the importance of the HPGA in broodiness in geese. However, within the HPGA, the mechanisms mediating broodiness in geese remain unclear.

When poultry birds become broody, they undergo multiple phenotypic changes, which encompass reproductive activities, folliculogenesis, ovulation, oviposition and incubation behaviors [3]. In the initial phase of the nesting process, the hierarchical follicles disappear gradually as no more follicles are recruited from the nonhierarchical pool. In the absolute incubation phase, both the hierarchical follicles and the nonhierarchical follicles vanish, and the geese externally manifest nesting behaviors, such as cessation of laying, a decrease in foraging time and fluffy feathers [12]. Therefore, the present study, considering the HPGA system as a whole, will assess the transcriptomic differences within goose HPGA tissues between the initial nesting and laying phases using next-generation sequencing and investigate possible mechanisms that may initiate the broodiness process in female geese.

## Materials and methods

### Ethics statement

All animal experimentation was approved by the Institutional Animal Care and Use Committee of Sichuan Agricultural University. The methods and protocols were carried out in accordance with the approved guidelines. All efforts were made to minimize suffering.

### Collection of tissue materials

**Fig 1:**
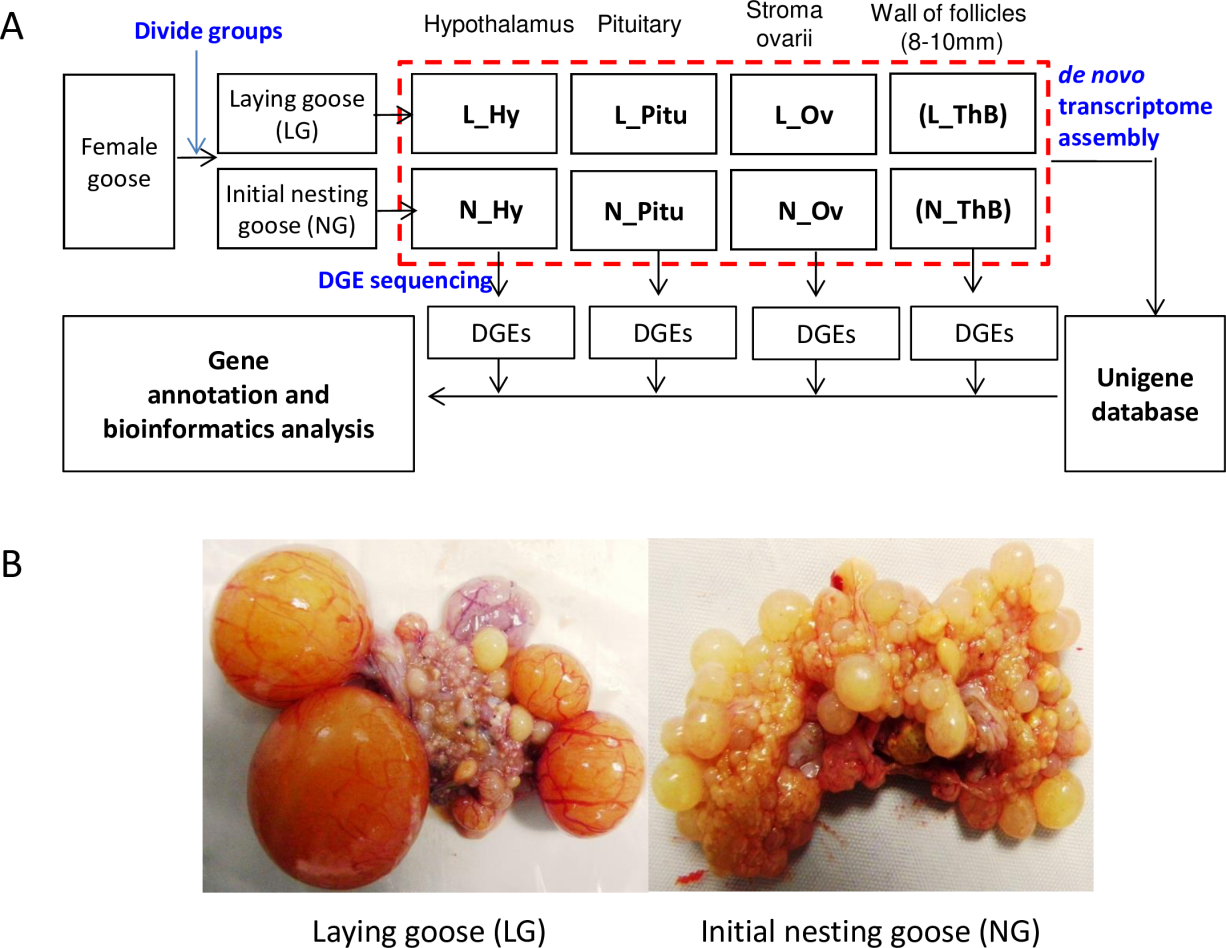
The entire experimental design and inclusion criteria for the groups of geese. A, The entire experimental design and sequencing procedure. L_Hy, L_Pitu, L_Ov and L_ThB, respectively, mean hypothalamus, pituitary gland, stroma ovarii and walls of follicles with a diameter range of 8–10 mm from laying geese. N_Hy, N_Pitu, N_Ov and N_ThB refer to the corresponding tissues from geese at the beginning of nesting. B, The standards for dividing geese into initial laying and nesting basing on the status of hierarchical follicles status (before the anatomical observation, the geese were distinguished first by their nesting behaviors, such as nesting, foraging times and fluffy feathers).

A healthy maternal line of Tianfu meat geese (*Anas cygnoides*, SICAU, Ya’an, China), 40±5 weeks of age and laying in regular sequences of at least 2–3 eggs, were used in the present study. The geese were provided feed and water *ad libitum* and were kept under natural temperature conditions. Supplemental artificial light was provided to allow 12 hr of daylight per day. Geese in the initial phase of the nesting process were first identified by their nesting behaviors, and further confirmation was obtained by anatomical observation of a lack of hierarchical follicles in the ovary (Fig1A). On the other hand, the laying geese were distinguished by the continuous sequence of ovulation and further confirmed by the presence of hierarchical follicles on the ovary (Fig 1B). Finally, a total of 6 laying and 6 initial nesting geese were selected and slaughtered, and tissues from the hypothalamus, the pituitary gland, the stroma ovarii, and the walls of nonhierarchical follicles (diameter range at 8–10 mm) were isolated and rapidly frozen in liquid nitrogen for RNA extraction.

### Total RNA extraction and *de novo* transcriptome assembly

Tissue samples from a total of 12 geese, including 6 nesting and 6 laying geese, were collected separately from the hypothalamus, the pituitary gland, the stroma ovarii, and the walls of nonhierarchical follicles (diameter range of 8–10 mm) and were pooled for RNA extraction. Total RNA was extracted by Trizol (Takara, Japan) according to the manufacturer’s protocol. The RNA quality and concentration was measured using the Qubit® RNA Assay Kit with the Qubit® 2.0 Fluorometer (Life Technologies, CA, USA) and the RNA Nano 6000 Assay Kit with the Agilent Bioanalyzer 2100 system (Agilent Technologies, CA, USA).

An equal amount of RNA per sample pool in each group, i.e., nesting and laying geese, was mixed together and used for unigene database construction. Sequencing libraries were generated using a NEBNext® Ultra™ RNA Library Prep Kit for Illumina® (NEB, USA), following the manufacturer’s recommendations. The library preparations were sequenced on an Illumina HiSeq 2500 platform, and 150 bp paired-end reads were generated. Clean reads were obtained by removing the reads containing adapters, reads containing poly-N and low-quality reads from the raw data. All the clean reads were assembled with the Trinity program [13], and all other parameters were set to default. Gene function was annotated based on the following databases: nr (NCBI non-redundant protein sequences), nt (NCBI non-redundant nucleotide sequences), Pfam (protein families), KOG/COG (Clusters of Orthologous Groups of proteins), Swiss-Prot (a manually annotated and reviewed protein sequence database), KO (KEGG Ortholog database), and GO (Gene Ontology).

### DEG sequencing and gene expression analysis in HPGA tissues

A total of 8 RNA samples, collected from 4 nesting and 4 laying geese, were extracted from HPGA tissues. The library construction for DEG sequencing was the same as described above. The library preparations were sequenced on an Illumina HiSeq 2500 platform, and 100 bp single-end reads were generated. Quantitative gene expression levels were estimated by RSEM [14] for each sample. First, clean data were mapped back onto the assembled transcriptome, and then the read count for each gene was obtained from the mapping results. Prior to differential gene expression analysis, the read counts for each sequenced library were adjusted with the edgeR program package through one scaling normalized factor. Differential expression analysis of the two samples was performed using the DEGseq (2010) R package [15]. P values were adjusted using q values [16]. Gene Ontology (GO) enrichment analysis of the DEGs was implemented by the GOseq R package based on Wallenius’ noncentral hypergeometric distribution [17], which can adjust for gene length bias in DEGs. KOBAS was used [18] to test the statistical enrichment of differential expression genes in KEGG pathways [19].

### qRT-PCR assay

Total RNA was extracted from each HPGA tissue of each individual goose in each group. First-strand cDNA was obtained using a cDNA synthesis kit (Takara, Dalian, China) according to the manufacturer’s protocols. The genes of interest were selected randomly among all DEGs. The glyceraldehyde-3-phosphate dehydrogenase gene (GAPDH) and 18S rRNA of geese were used as internal controls. The primers were designed using the Primer 5 software (Primer Biosoft International, USA); S1 Table lists information on all the primers. qRT-PCR was conducted using a 96-well iCyclerIQ5 (USA) and a TaKaRa Ex Taq RT-PCR kit (Takara, Dalian, China). The procedure was as follows: 10 s of pre-denaturation at 95°C, followed by 45 cycles of 95 and 62°C for 5 and 30 s, respectively. All reactions were run in triplicate. The relative mRNA expression levels were calculated using the normalized relative quantification method followed by 2^−ΔΔCT^ [20].

## Results and analysis

### Transcriptome sequencing, unigene assembly and annotation

A mixed cDNA sample representing 8 kinds of HPGA-related tissues, derived from groups of geese at two different stages of the reproductive cycle, was prepared as a cDNA library for sequencing. This sequencing run produced 42.3 million raw reads and 37.0 million clean reads for each paired end. Assembly of all the clean reads produced a clean sequence of 11.1 Gb, which formed 160, 801 transcripts (Table 1). The longest transcript of each gene was considered a unigene; consequently, 128 148 unigenes were selected among the total transcripts. The transcripts ranged in size from 201 to 15,495 bp, with an average size of 915 bp, whereas the Unigenes ranged in size from 201 to 15,495 bp with an average size of 691 bp (S1A & S1B Fig). All unigenes were compared against the public nr, GO, KO, PFAM, Swiss-Prot, KOG, and NT databases individually to annotate them with relevant gene information. All selected unigene sequences were annotated, and 29.64% (37,984) of them were annotated in at least one database (Table 2 & S2 Table). The lengths of the peptides BLASTed against the nr, Swiss-Prot and KEGG databases were mainly distributed in the range of approximately 80 to 1,000 amino acid residues, whereas the peptides predicted by ESTScan were mainly distributed in the range of approximately 40 to 600 amino acid residues (S1C & S1D Fig).

**Table 1:** Quality analyses of transcriptome sequencing quality.

**Items**	**Reads-1**	**Reads-2**
Raw reads	42,316,668	42,316,668
Clean reads (percentage)	37,003,710 (87.44%)	37,003,710 (87.44%)
Containing N (percentage)	47,244 (0.11%)	47,244 (0.11%)
Low quantity (percentage)	4,820,799 (11.39%)	4,820,799 (11.39%)
Adapter related (percentage)	444,915 (1.05%)	444,915 (1.05%)
Clean bases	5.55 G	5.55 G
Error rate	0.06%	0.06%
Q20	97.26%	95.25%
Q30	91.67%	87.36%
GC Content	48.19%	48.14%

Note: Q20 represents an error rate less than 0.01, and Q30 represents an error rate less than 0.001. “Reads-1” presents the left-end reads, and “Reads-2” presents the right-end reads.

**Table 2:** Size distribution of valid unigenes compared with the public databases.

	Number of unigenes	Percentage (%)
Annotated in nr	23,570	18.39
Annotated in nt	29,607	23.1
Annotated in KO	8,766	6.84
Annotated in Swiss-Prot	21,456	16.74
Annotated in Pfam	21,856	17.05
Annotated in GO	24,334	18.98
Annotated in KOG	11,739	9.16
Annotated in all databases	4,799	3.74
Annotated in at least one database	37,984	29.64
Total unigenes	128,148	100

### DEG sequencing and gene expression analysis in the HPGA

In each group, the transcriptome was determined by DEG sequencing in the hypothalamus, the pituitary gland, the stroma ovarii, and the wall of nonhierarchical follicles (diameter range of 8–10 mm). The number of raw reads generated was in the range of 15.2 to 18.7 million in all libraries. More than 97% of the raw reads were filtered as clean reads. The number of clean reads mapped to the constructed unigene database range between 14.9 and 18.3 million in all libraries, and more than 80% were perfectly matched (shown in Table 3).

**Table 3:** Quality analyses of DEG Library.

**Items**	**L_Hy**	**N_Hy**	**L_Pitu**	**N_Pitu**	**L_Ov**	**N_Ov**	**L_ThB**	**N_ ThB**
Raw reads	15,756,265	16,199,669	15,250,413	18,743,038	17,041,444	16,108,018	17,170,334	17,882,213
Clean reads(Percentage,%)	15,456,165(98.10)	15,875,322(98.10)	14,960,583(98.10)	18,395,025(98.14)	16,714,680(98.08)	15,779,535(97.96)	16,861,634(98.20)	17,539,269(98.08)
Containing N(Percentage,%)	254 (0.00)	258 (0.00)	241(0.00)	315(0.00)	262(0.00)	250(0.00)	272(0.00)	257(0.00)
Low quantity(Percentage,%)	292,992(1.86)	310,970(1.92)	282,651(1.85)	335,304(1.79)	319,778(1.88)	319,864(1.99)	301,920(1.76)	332,274(1.86)
Adapter related(Percentage,%)	6,854(0.04)	13,119(0.08%)	6,938(0.05)	12,394(0.07)	6,724(0.04)	8,369(0.05)	6,508(0.04)	10,413(0.06)
Q20	96.46%	96.36	96.53	96.54	96.45	96.38	96.52	96.39
Q30	89.80%	89.59	90.06	90.04	89.83	89.71	89.94	89.69
GC content	48.62%	49.04	48.33	48.41	48.83	48.73	48.75	49.12
Total mapped(Percentage,%)	13,655,973(88.35)	14,035,547(88.41)	12,974,226(86.72)	15,851,802(86.17)	14,664,056(87.73)	14,036,061(88.95)	14,972,499(88.80)	15,511,948(88.44)

**Fig 2:**
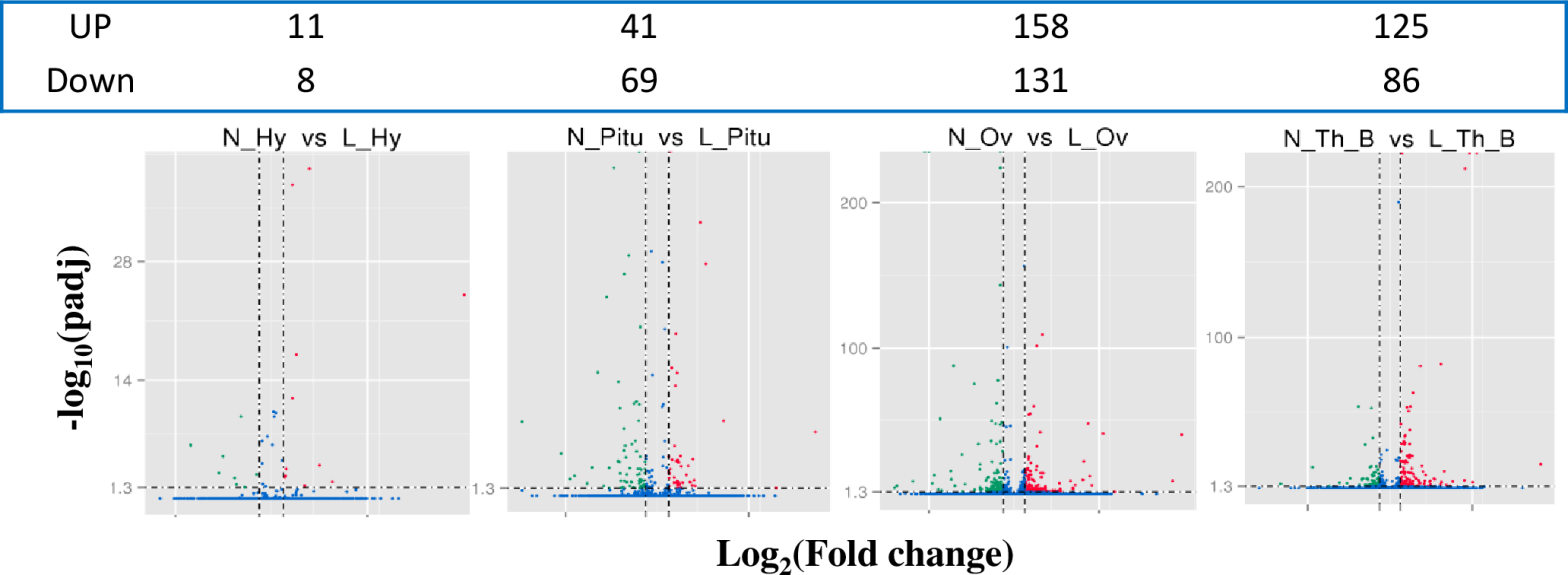
Volcano map of differential gene expression. The horizontal coordinates represent the fold changes of genes between the laying and initial nesting groups. The longitudinal coordinates represent the statistical significance of the changes in gene expression. The smaller the p value, the greater the value of -log_10_p. Each dot in the image represents one gene; the blue ones indicate no significant difference in the gene, while the red ones indicate a significant difference. All the DEGs enriched in the hypothalamus, pituitary gland, stroma ovarii and walls of follicles with diameters of 8–10 mm between laying geese and early nesting geese are provided in S3–S6 Tables.

**Fig 3:**
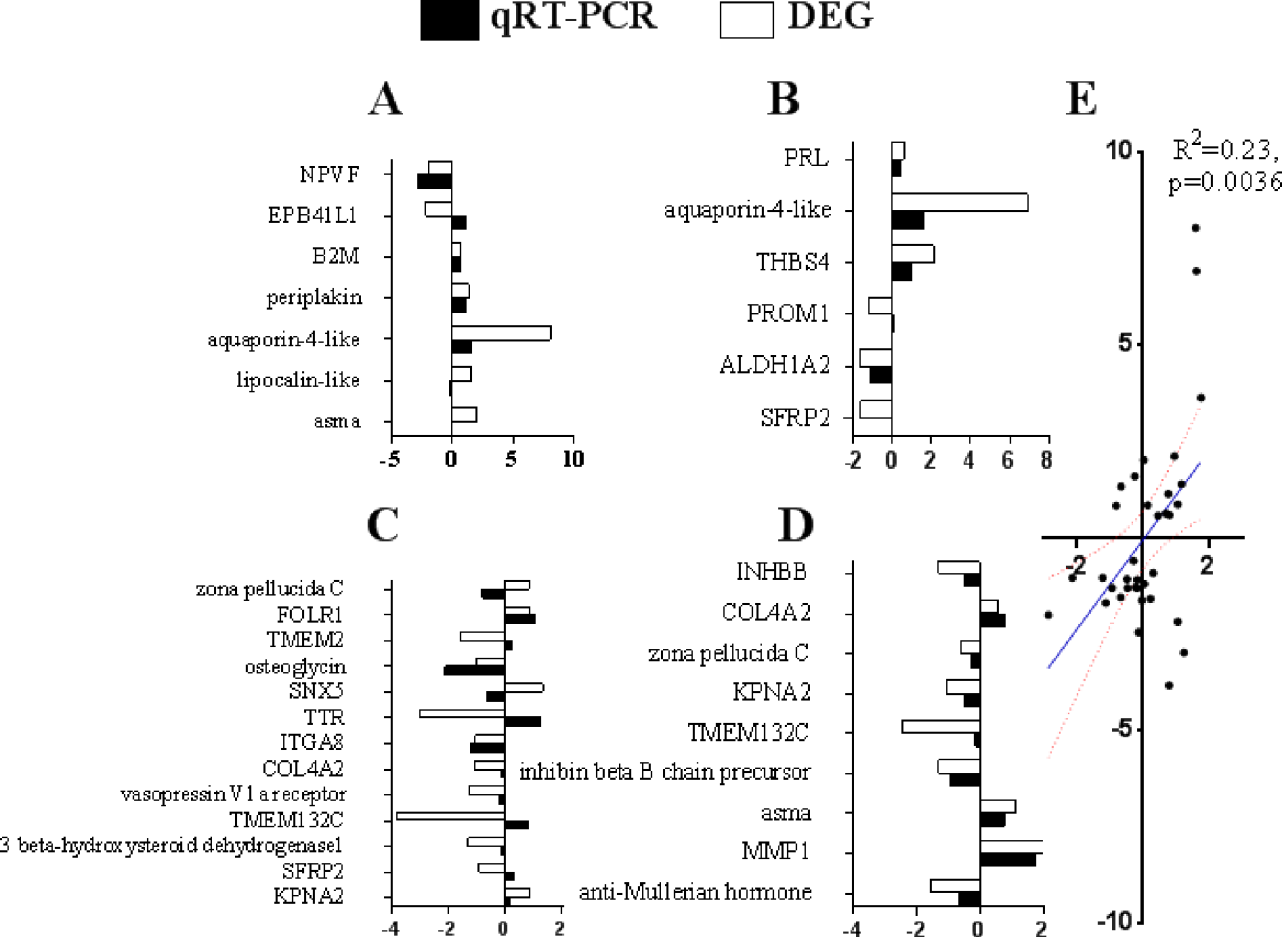
Validation of the sequencing data by qRT-PCR. A-D, Validation of results for the hypothalamus, the pituitary gland, the stroma ovarii and the walls of follicles (diameter range 8–10 mm), respectively. E, The correlation coefficient between qRT-PCR and DEG data was analyzed based on all validated genes. The genes were selected randomly from S3–S6 Tables, and the primers designed for qRT-PCR are listed in S1 Table. For both the qRT-PCR and DEG results, the fold changes were calculated based on the expression levels in laying and nesting group and were then converted to log_2_(Fold change).

The clean reads of each DEG library were mapped to the reference database (unigene database) to generate a read count value. Then, Reads Per Kilobase per Million mapped reads (RPKM) were utilized to quantify the expression level of each gene. The correlation coefficient of gene expression levels among samples indicated the high relevance and reliability of the experimental sample selection in the present model (S2 Fig). The RPKM boxplot showed that clear differences existed in gene expression between the comparison groups (S3 Fig). To enrich the genes with a significant difference in expression between the Laying and Nesting groups, we calculated the gene expression levels using the RPKM with a default value of |log_2_(FoldChange)|>1 and q value<0.05. Ultimately, we found that 19, 110, 289 and 211 genes were enriched in the hypothalamus, the pituitary gland, the stroma ovarii and the walls of nonhierarchical follicles, respectively (Fig 2). The Venn diagram illustrates the number of common DEGs among comparison groups, as well as the number of specific DEGs in each comparison group. As shown by the Venn diagram (S4 Fig), there were 10, 80, 213, and 136 DEGs identified between comparison groups in the hypothalamus, the pituitary gland, the ovary and the nonhierarchical follicle walls, respectively. We selected at least 6 DEGs in each comparison group to validate the sequencing data by determining mRNA expression using qRT-PCR. The data showed that all the validated genes contributed a high correlation coefficient index between qRT-PCR and sequencing data (Fig 3).

According to the GO classification for the comparison groups L_Hy and N_Hy (S5 Fig), most of the DEGs are associated with biological process (BP) terms; among cellular component (CC) terms, only terms regarding the extracellular region reached significance. For the comparison groups of L_Pitu and N_Pitu, the top 30 highly enriched GO terms were identified, and most DEGs were classified under BP terms, but none of them exhibited significant differences. For the comparison groups of L_Ov and N_Ov, there were many BP, CC and molecular function (MF) terms that were enriched, and all of the top 30 enriched GO terms reached significance. Similarly, for the comparison groups of L_ThB and N_ThB, all of the top 30 enriched GO terms reached significance, and most of the enriched GO terms were classified as BP terms.

**Fig 4:**
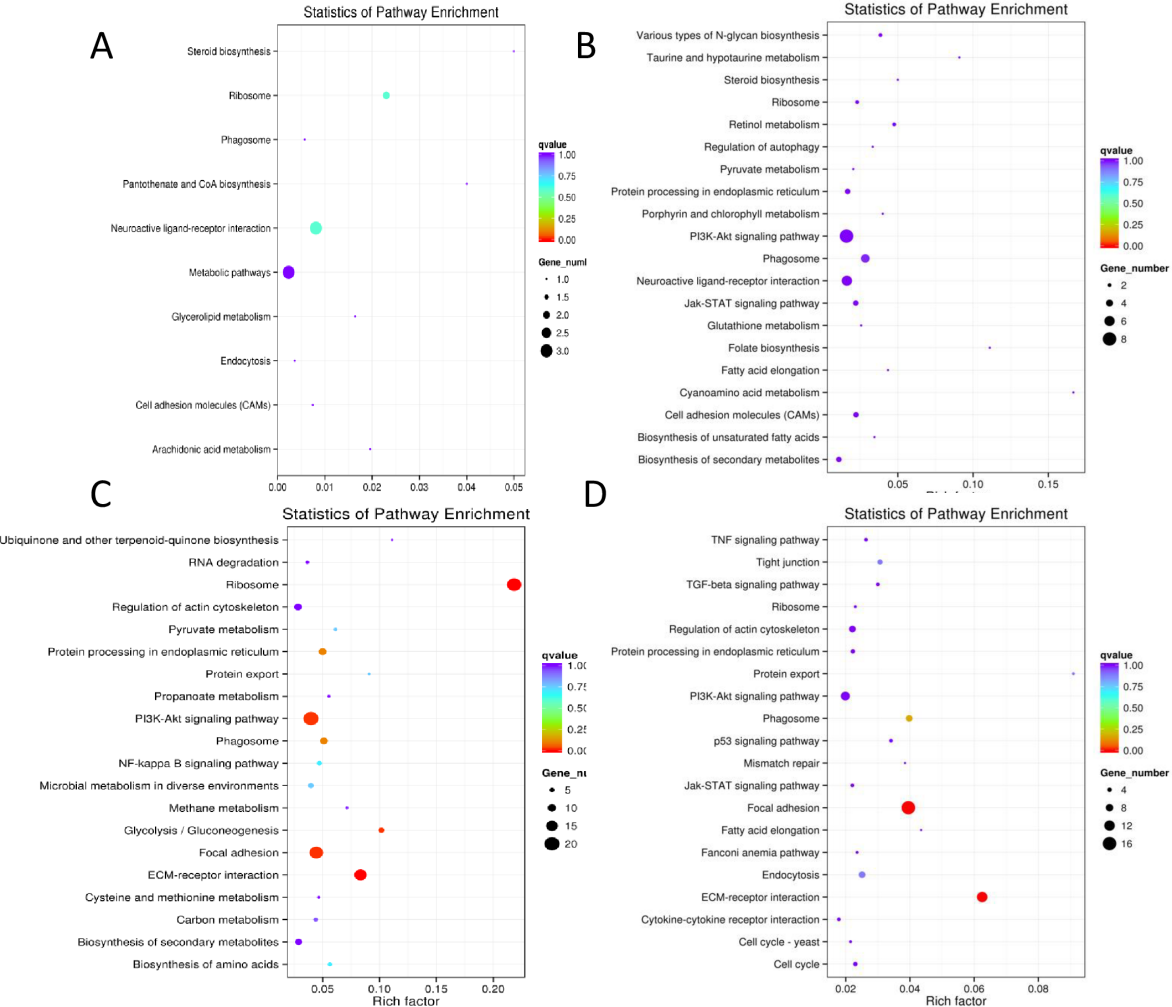
KEGG enrichment scatter plot. A-D, The enriched pathways based on DEGs distributed in the hypothalamus, the pituitary gland, the stroma ovarii and the walls of follicles (diameter range 8–10 mm); the top 20 enriched pathways, sorted by enrichment factor, in each comparison group. The enrichment factor was the ratio of the number of DEGs enriched in the pathway to the total number of all annotated genes enriched in this pathway.

By BLASTing the DEGs against the KEGG database, we enriched pathways that might be involved in initiating the nesting process in geese (Fig 4). The top 20 significantly enriched pathways in each HPGA tissue were listed in Fig 4. In the hypothalamus, metabolic pathways accounted for the largest number of DEGs, and steroid biosynthesis had the largest enrichment factor. In the pituitary gland, the PI3K-AKT and neuroactive ligand-receptor interaction pathways have the largest number of DEGs, cyanoamino acid metabolism had the largest enrichment factor. In the stroma ovarii, the ribosome, ECM-receptor interaction and focal adhesion pathways had the largest numbers of DEGs and the highest enrichment factors, and they also reached significant difference levels. In the follicle walls, ECM-receptor interaction and focal adhesion had the largest enrichment factor and reached significant difference levels.

### The key molecules within the HPGA that may initiate broodiness in female geese

To investigate the possible regulatory pathways involving the HPGA that may initiate the nesting process in geese, the present study focused on the secreted proteins in the hypothalamus, the receptor genes and secreted proteins in the pituitary gland, and the genes and proteins in the stroma ovarii and follicle walls that interact with the secreted proteins from the hypothalamus and the pituitary gland. In the hypothalamus, 5 DEGs encoding secreted proteins were selected; they included 4 DEGs that were down-regulated and 1 that was up-regulated in the nesting geese (Table 4). Interestingly, HCRT was down-regulated in nesting geese. HCRT plays a significant role in the regulation of food intake [21] and the sleep-wake cycle [22], possibly by coordinating the complex behavioral and physiological responses of these complementary homeostatic functions. In the pituitary gland, 8 DEGs were enriched; they encoded proteins that could interact with the secreted proteins from the hypothalamus (Table 4). Among them, PRL, FSH, LHB and GH have well-understood potential roles in poultry broodiness [1, 12]. In addition, a total of 17 DEGs encoding secreted proteins were identified among the 110 DEGs. Among those 17, genes such as RLN3 [23] and GDNF [24] have been reported to modulate the reproductive process in animals. The ovary and follicular walls are the target organ and tissues for the secreted proteins transported in the blood from the hypothalamus and the pituitary gland. Therefore, the current experiments investigated the interaction of DEG products with the secreted proteins from the hypothalamus and the pituitary gland. Among the DEGs, there were 28 and 34 enriched in the ovary and the follicular walls, respectively (Table 4).

**Table 4:** The DEGs of interest that enriched in HPGA-related tissues.

Tissue	Type	DEG name
Hypothalamus	SP	HCRT↓, Npvf↓, OXT↓, B2M↓, POMC↑
Pituitary	TP	GCGR↑, LDLRAP1↑, HMOX1↑
	SP&TP	Crh↓, FSH↓, PRL↑, LHB↓, GH↓
	SP	CLEC3B↓, MGP↓, NOELIN-2↑, CHRDL1↑, QPCT↓, VCAN↓, RLN3↓, SFRP2↓, TFPI↑, TIMP2↑, CEL↓, GDNF↓
Ovary	TP	AVPR1A↓, B2M↑, BAMBI↑, CACNA1H↓, CALR↑, CCL21↑, CHGB↑, DBI↑, EOMES↑, Fbln2↓, FBN1↓, Fn1↓, Fos↓, HSD3B1↓, IL13RA1↓, LUM↑, MFGE8↓, MGLL↓, Nid1↓, Ogn↓, Ppif↑, Rrbp1↓, Stat1↑, TAR1↓, THBS1↓, TTR↓, VCAM1↑, VWF↓
Follicle wall	TP	Apelin↑, CD44↑, Cish↑, Ctgf↑, CYP1B1↑, EGR1↑, F3↑, Fbn1↑, Fn1↑, Furin↓, GBP1↑, Hspg2↑, Htr2a↑, Large↓, MGLL↓, Mmp1↑, Mmp27↑, Mmp3↑, Mmp9↑, MYLK↑, OGN↑, PCSK7↓, Pdlim5↓, Procr↓, Rab7a↓, Rrbp1↑, SHH↓, SLC2A3L↑, Socs3↑, STAT3↑, TAR1↑, TGM2↑, THBS1↑, TOP2A↓

Note: The DEGs coding for secreted proteins (SPs) in the hypothalamus and pituitary gland were identified by checking their information in the UniProt database. The target proteins (TP) in the pituitary gland, ovary and follicle wall that can interact with the secreted proteins from the hypothalamus and pituitary gland were identified using Cytoscape software and the STRING database (http://string-db.org/). An upward-pointing arrow after a gene name represents up-regulation of the gene in nesting geese compared with laying geese, while a downward-pointing arrow means down-regulation of the gene.

**Fig 5:**
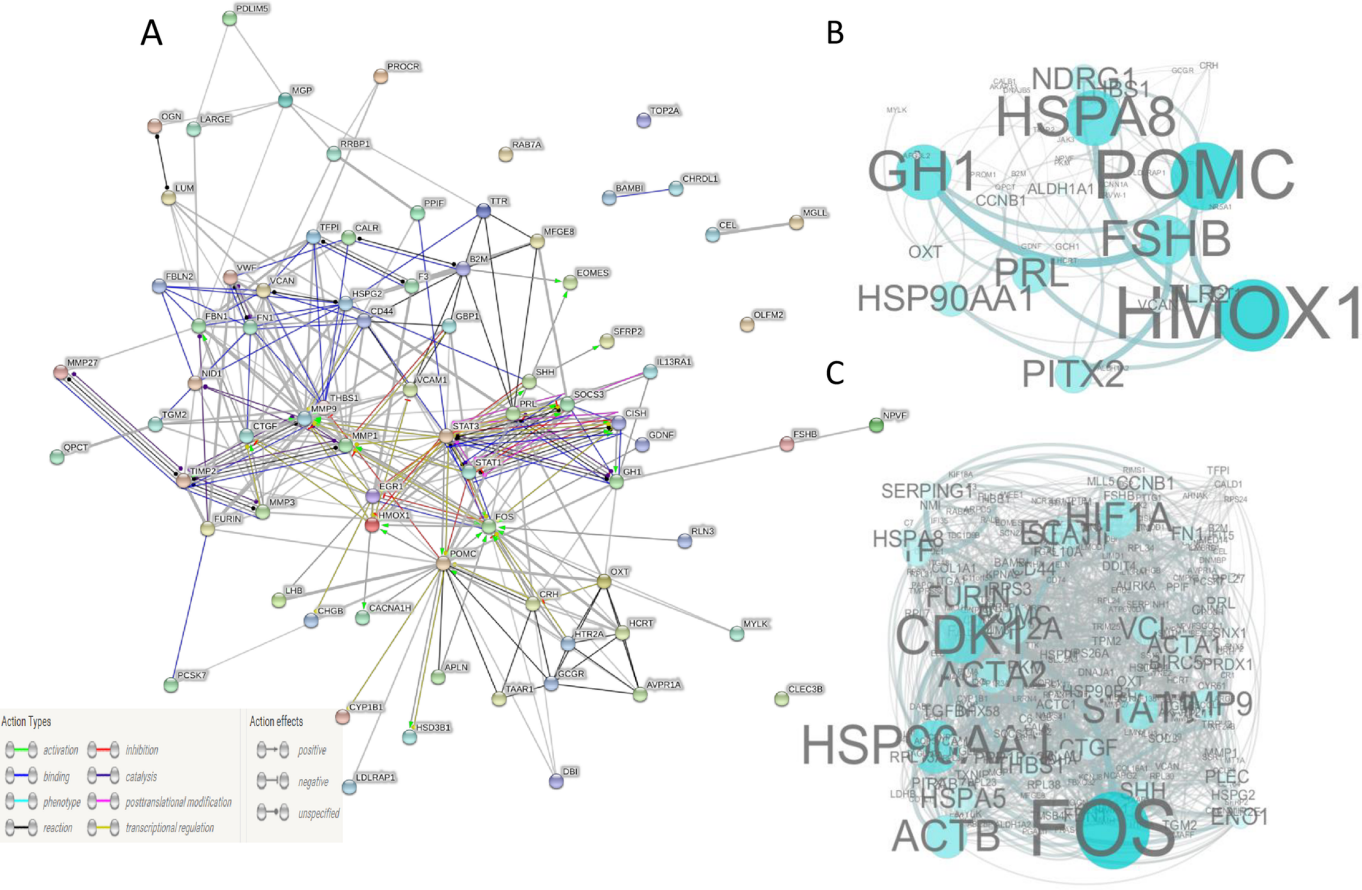
Protein-protein interaction relationships based on the DEGs. A, Table 4 lists the DEGs that were analyzed. B, Networks are based on the DEGs that code for secreted proteins in the hypothalamus (Table 4) and all DEGs from the pituitary gland (S4 Table). C, Networks based on the DEGs that code for secreted proteins in the hypothalamus and pituitary gland (Table 4) and all DEGs in the stroma ovarii and follicle wall (S4 and S6 Tables). The information on the interactions between the proteins was downloaded from the STRING database (http://string-db.org/).

All the proteins listed in Table 4 were further analyzed to generate protein-protein interaction networks, and the results are presented in full in Fig 5A. Fig 5B shows their positions in the regulatory networks as depicted by Cytoscape. POMC, HMOX1, HSPA8, FSHB, GH and PRL seemingly occupied the core regulatory positions in the networks, and they may play central roles in mediating the functions of other molecules function in the broodiness process in geese. The secreted proteins from the hypothalamus and the pituitary gland were then enriched among the DEG products from the ovaries and follicular walls. Fig 5C shows their positions in the regulatory networks, and it seems that FOS, HSP90AA, and CDK1 were in the core regulatory positions in the networks modulating the initiation of broodiness in geese.

**Fig 6:**
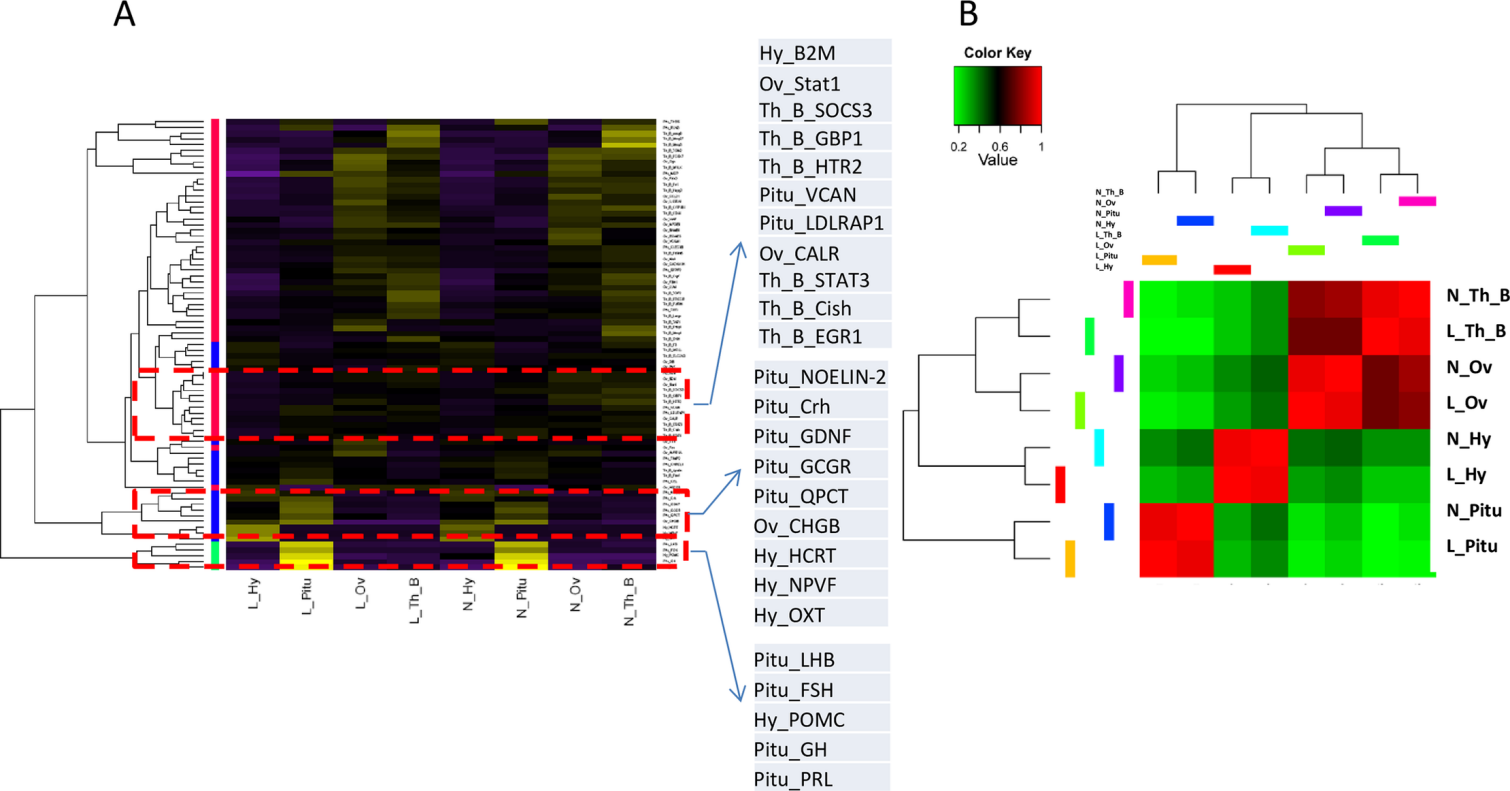
Hierarchical clusters of DEGs. A, Clustering of the DEGs listed in Table 4. B, Clustering of groups based on gene expression patterns among groups.

These DEGs (listed in Table 4) identified in the present study as enriched in HPGA tissues were clustered by their gene expression levels (Fig 6). Some of them form especially close clusters in terms of their expression patterns across the HPGA tissues; the three closest clusters were designated 1, 2, and 3. Cluster 1 includes the genes POMC in the hypothalamus and LHB, FSH, GH and PRL in the pituitary gland. Cluster 2 includes the genes HCRT, NPVF and OXT in the hypothalamus; NOELIN-2, Crh, GDNF, GCGR and QPCT in the pituitary gland; and CHGB in the ovary tissues. Cluster 3 includes the genes B2M in the hypothalamus; VCAN and LDLRAP1 in the pituitary gland; Stat1 and CALR in the ovary tissues’ and SOCS3, GBP1, HTR2, STAT3, Cish and EGR1 in the follicular walls. The clustering of these genes by expression patterns indicates that regulatory relationships may exist among them. Parts of the clusters support the protein-protein interaction relationships shown in Fig 5A, i.e., POMC and LHB, HCRT, OXT, GCGR and CRH.

## Discussion

The HPGA plays central role in mediating not only the reproductive systems but also the immune systems of poultry [25]. Here, we tried to investigate the relevant molecular networks involving the HPGA-related tissues. In this study, 19, 110, 289, and 211 DEGs were found to be enriched in the hypothalamus, the pituitary gland, the stroma ovarii, and the walls of nonhierarchical follicles, respectively. It is well known that several classical regulatory networks exist in the HPGA to modulate the animal reproductive system. The roles of GnRH, VIP, PRL, LH and FSH in poultry broodiness have been well described [26]. In detail, environmental conditions can activate hypothalamic release of GnRH and VIP peptides, which reach the pituitary cells through the hypophyseal portal vessels. The gonadotrophs in the pituitary gland, controlled by GnRH, release LH and FSH, while lactotrophs in the pituitary gland, stimulated by VIP, are involved in the synthesis of PRL. In females, FSH promotes gonadal maturation and follicular selection and regulates progesterone secretion by the granulosa cells of the pre-hierarchical follicles. LH controls estrogen and androgen production by mature ovarian follicles[1][12, 26]. In the present study, the expression levels of FSH and LH were down-regulated, and the PRL was up-regulated in the pituitary glands of nesting geese. Our findings were consistent with the above mentioned reports, supporting the idea that molecules such as PRL, FSH and LH that may initiate and sustain poultry broodiness. In addition, many studies have discussed the contributions of these molecules to classical regulatory pathways within the HPGA. However, changes in GnRH expression in the hypothalamus were not observed during current study indicating GnRH was possible not the key molecules in initiating broodiness in geese.

Some other new molecules may also be involved in the broodiness process in geese, such as OXT, CHRDL1 and GH. OXT, shown in other model systems to the contraction of the smooth muscle of the uterus and the mammary gland, was down-regulated in the hypothalamus of nesting geese, which was previously reported to specifically bind oxytocin [27], and these results indicate there is a role of OXT in the reproductive system; however, no clear mechanism has been reported for its roles in poultry broodiness. CHRDL1 was reported to function as a BMP4 antagonist by binding to it and preventing its interaction with receptors[28], and BMP4 can affect poultry follicular development [29]. In the present study, the results indicates that CHRDL1 was up-regulated in the pituitary glands of nesting geese, which suggests the role of CHRDL1 in inhibiting follicular development through the HPGA.

Reduction of appetite is possibly the first step and a prerequisite for initiating broodiness in poultry. When poultry are in the nesting phase, they often display a number of accompanying behaviors such as reduction of foraging time, loss of feathers from the breast to form a brood patch and even cessation of egg laying[12]. It can be concluded that if poultry spend most of their time on feeding and foraging, then broodiness will be delayed. We have identified two relevant genes that are differentially expressed in the hypothalamus in the early phase of broodiness in geese.

HCRT has been reported to have a role in the regulation of food intake and the sleep-wake cycle [21, 22], and POMC can increase the sense of stomach fullness and have a suppressive effect on appetite [30]. Our data showed that HCRT and POMC were down- and up-regulated, respectively, in the hypothalamus of nesting geese, which indicated that these two genes may have a coordinated function in reducing the appetite of nesting geese. Additionally, these two genes also have direct or indirect roles in affecting the classical molecules involved in broodiness in geese, such as FSH, PRL, LHB and GH, and some steroid synthesis-related genes, such as Cyp1B1 and hsd3b1 (Fig 5A), and they were also clustered together by their expression patterns (Fig 6), indicating their integrated role in the behavior and follicular development of geese. These findings also imply a possible new strategy of increasing the appetite possibly through administration of HCRT of poultry to reduce broodiness.

Some new core regulatory molecules within the HPGA were identified as factors controlling broodiness in geese. POMC, HMOX1 and HSPA8 emerged as new core regulatory molecules, aside from the classical molecules FSHB, GH and PRL that operate on the regulatory networks between the hypothalamus and the pituitary gland. HMOX1 was up-regulated in the nesting geese. Fig 5A shows that HMOX1 can be activated by POMC, which is a secreted protein and is up-regulated in the hypothalamus. HMOX1 can cleave the heme ring at the alpha methylene bridge to form biliverdin [31]; however, there have been no studies focused on its roles in the reproductive system. FOS, HSP90AA, and CDK1 were in the core regulatory positions in the networks between the secreted proteins from the hypothalamus and pituitary gland and all DEGs in glandular tissues. FOS is thought to have important roles in signal transduction, cell proliferation and differentiation, and it has been widely studied in relation to ovarian cancer and steroid hormone synthesis in the ovaries[32, 33].

The DEGs in the ovaries and follicle walls relating to follicular development and steroid hormone synthesis may be involved in initiating broodiness in geese. Studies by Xu et al. reported that the genes relating to steroid hormone synthesis were identified as differentially expressed in the ovary tissues between laying and broodiness in geese [34, 35]. Our data showed that the enriched DEGs interacting with the secreted proteins from the hypothalamus and pituitary included 28 and 34 genes in the ovaries and the follicular walls, respectively. Of them, genes including Mmps (Mmp1, Mmp27, Mmp3 and Mmp9)[36], apelin[37], EGR1[38], Hspg2[39] and Procr [40] are reportedly associated with follicular development, and genes including CYP1B1 [41] and HSD3B1[42] are involved in steroid hormone synthesis. These data imply that the ovaries and the follicle walls respond to the regulation of secreted proteins from hypothalamus and pituitary gland during the nesting process in geese.

**Fig 7:**
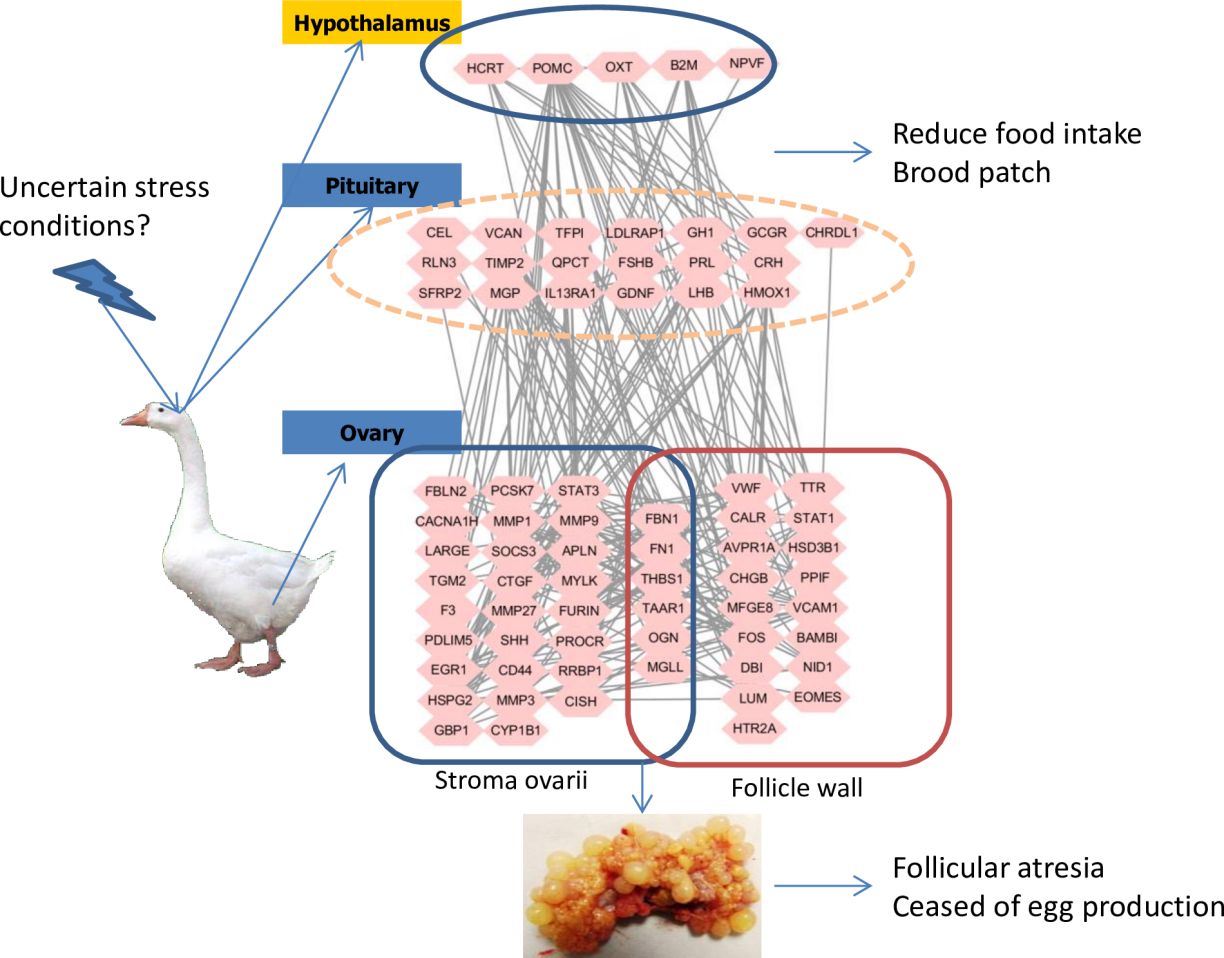
Regulatory processes within the HPGA that may take part in initiating broodiness in geese.

In the regulatory process within HPGA that initiates broodiness in geese (Fig 7), there has been some uncertainty about which external conditions stimulate the hypothalamus of geese to change the expression of secreted proteins [12]. The present results indicate that appetite-related genes, including HCRT and POMC, may play essential roles in this phase. These changes are consistent with the observed reduction of foraging time in the early phase of broodiness in geese. The relevant external conditions remain uncertain, although some studies have proposed that such conditions include the availability of a quiet nest site, minimal disturbance and the accumulation of a clutch of eggs [12]. Additionally, the secreted proteins from the hypothalamus reach the pituitary cells through the hypophyseal portal vessels and cause the changes in gene expression level in pituitary cells. Aside from the classical pathways of PRL, FSH and LH, the current experiment demonstrated that OXT, CHRDL1 and GH are new molecules in the pituitary gland that may be involved in broodiness in geese. HMOX1 may play a role in integrating signals from the hypothalamus and inducing gene expression in the pituitary gland. Finally, the proteins secreted by the hypothalamus and the pituitary gland reach the ovaries and follicular tissues via blood vessels, and they interact with their target proteins to suppress follicular development and change the rate of steroid hormone synthesis. The results of the present study demonstrated that FOS, HSP90AA and CDK1 are in the core regulatory positions of the interaction network and integrate the signals from the hypothalamus and the pituitary gland to control gene expression in the ovaries and follicles.

## Conclusion

In this study, a unigene database was constructed for gene identification within HPGA-related tissues by transcriptome sequencing, and 19, 110, 289, and 211 DEGs were identified in the hypothalamus, the pituitary gland, the stroma ovarii, and the walls of nonhierarchical follicles, respectively, between laying and nesting geese. Appetite reduction may be the first step and a prerequisite for initiating broodiness in geese. OXT, CHRDL1 and GH expressed in the pituitary gland may be involved in broodiness in geese. HMOX1 may play a role in the pituitary gland, and FOS, HSP90AA and CDK1 may play a role in the ovaries and follicles; moreover, all these genes may consolidate and transduce signals that regulate the HPGA during broodiness in geese.

## Supporting information

S1 Fig Frequency distributions of the assembled transcripts, unigenes, and predicted peptides. A, Length frequency distribution of transcripts. B, Length frequency distribution of unigenes. When the lengths of the transcripts are added together one by one, from the longest to the shortest, N50 (N90) is defined as the length of the transcript at which the accumulated length reaches no less than 50% (90%) of the total length. C, Length frequency distribution of peptides predicted by BLAST. D, Length frequency distribution of peptides predicted by ESTScan software.
(TIF)

S2 Fig Correlation of gene expression levels among samples. A, Correlation coefficient with R^2^ value. B, Clusters of groups by correlation coefficient.
(TIF)

S3 Fig Boxplot showing RPKM (Reads Per Kilobase per Million mapped reads) distributions among groups. The horizontal coordinate is sample group, and the vertical coordinate is log_10_(RPKM+1). Each column of the boxplot contains 5 statistics (from top to bottom, the maximum value, the third quartile, the median, the first quartile and the minimum value).
(TIFF)

S4 Fig Venn diagram showing the differentially expressed genes among groups. The numbers in each large circle represent the total number of differentially expressed genes (DEGs) for each tissue within the HPGA, and the numbers in the overlapping portions of the circles represent genes that are shared between groups.
(TIF)

S5 Fig GO analyses of DEGs in each tissue of the HPGA. A-D, GO terms distributed in the hypothalamus, the pituitary gland, the stroma ovarii and the walls of follicles (diameter range 8–10 mm). The top 30 GO terms for each comparison group are listed. The GO terms were sorted by the ratio of the number of DEGs enriched in the GO term to the total number of annotated genes enriched in this GO term. All GO terms enriched by the DEGs in each HPGA tissue are provided in S7–S10 Tables.
(TIF)

S1 Table Primers for real-time PCR. (DOCX)

S2 Table Annotation of all unigenes identified in this study. (XLS)

S3 Table DEGs enriched between N_Hy and L_Hy. (XLS)

S4 Table DEGs enriched between N_Pitu and L_Pitu. (XLS)

S5 Table DEGs enriched between N_Ov and L_Ov. (XLS)

S6 Table DEGs enriched between N_ThB and L_ThB. (XLS)

S7 Table GO terms enriched between N_Hy and L_Hy. (XLS)

S8 Table GO terms enriched between N_Pitu and L_Pitu. (XLS)

S9 Table GO terms enriched between N_Ov and L_Ov. (XLS)

S10 Table GO terms enriched between N_ThB and L_ThB. (XLS)
