# Supplementary figures and images for: Transcriptome analysis revealed the possible regulatory pathways initiating female geese broodiness within the hypothalamic-pituitary-gonadal axis
Source: PLoS One. 2018 Feb 6;13(2):e0191213. doi: 10.1371/journal.pone.0191213 (PMC5800542; doi:10.1371/journal.pone.0191213)

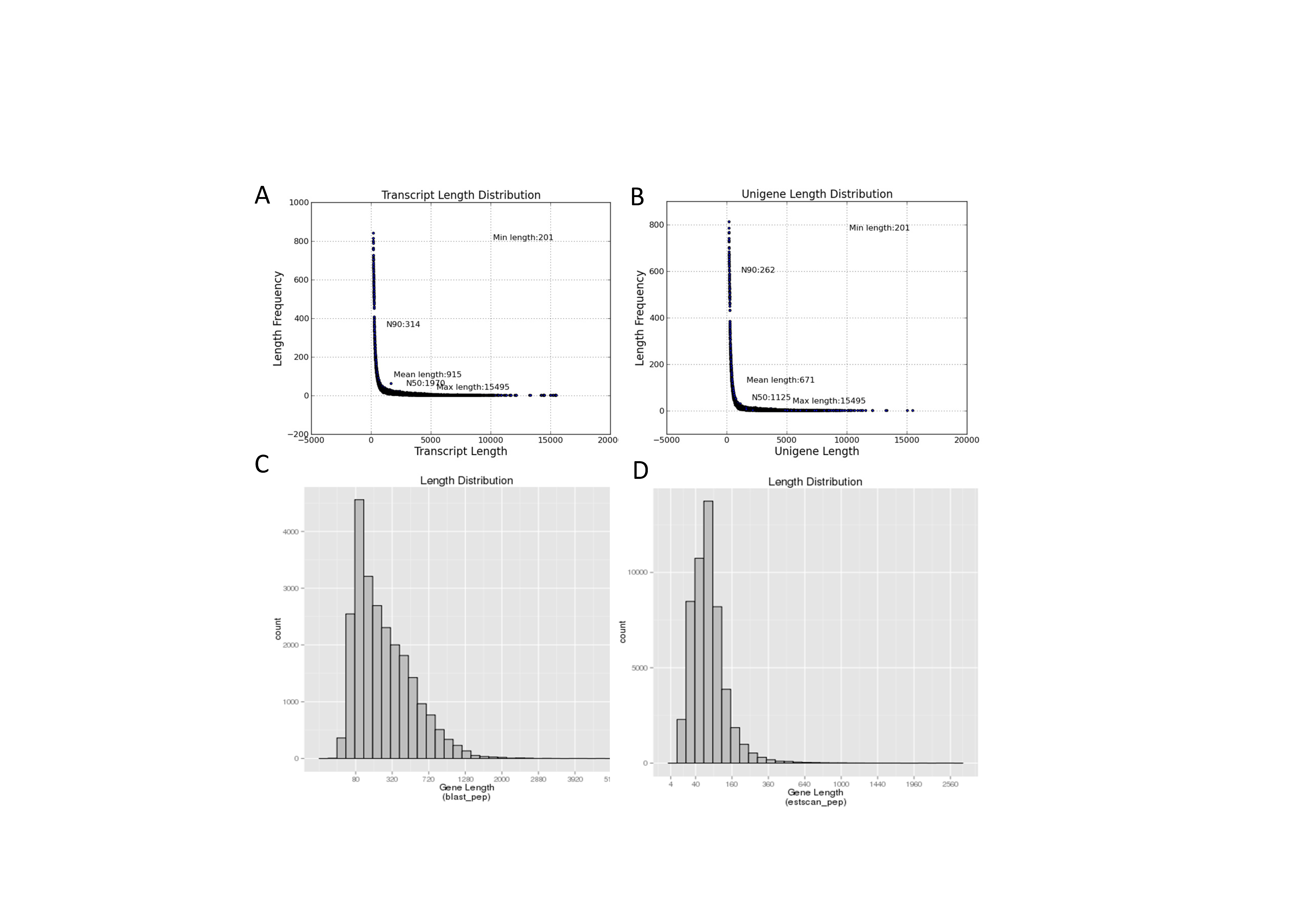

Supplement: S1 Fig — A, Length frequency distribution of transcripts. B, Length frequency distribution of unigenes. When the lengths of the transcripts are added together one by one, from the longest to the shortest, N50 (N90) is defined as the length of the transcript at which the accumulated length reaches no less than 50% (90%) of the total length. C, Length frequency distribution of peptides predicted by BLAST. D, Length frequency distribution of peptides predicted by ESTScan software. (TIF) [file pone.0191213.s001.tif]

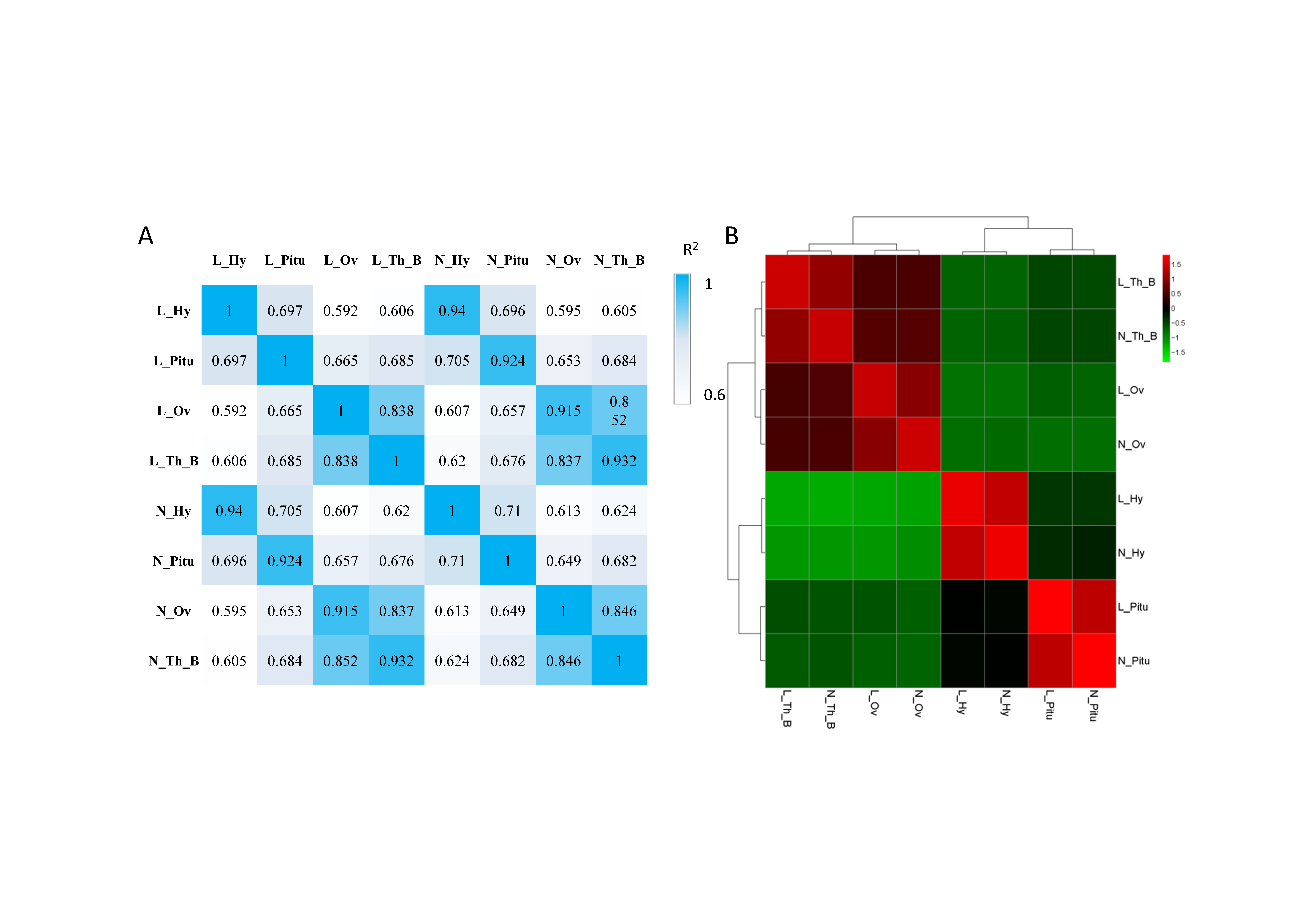

Supplement: S2 Fig — A, Correlation coefficient with R2 value. B, Clusters of groups by correlation coefficient. (TIF) [file pone.0191213.s002.tif]

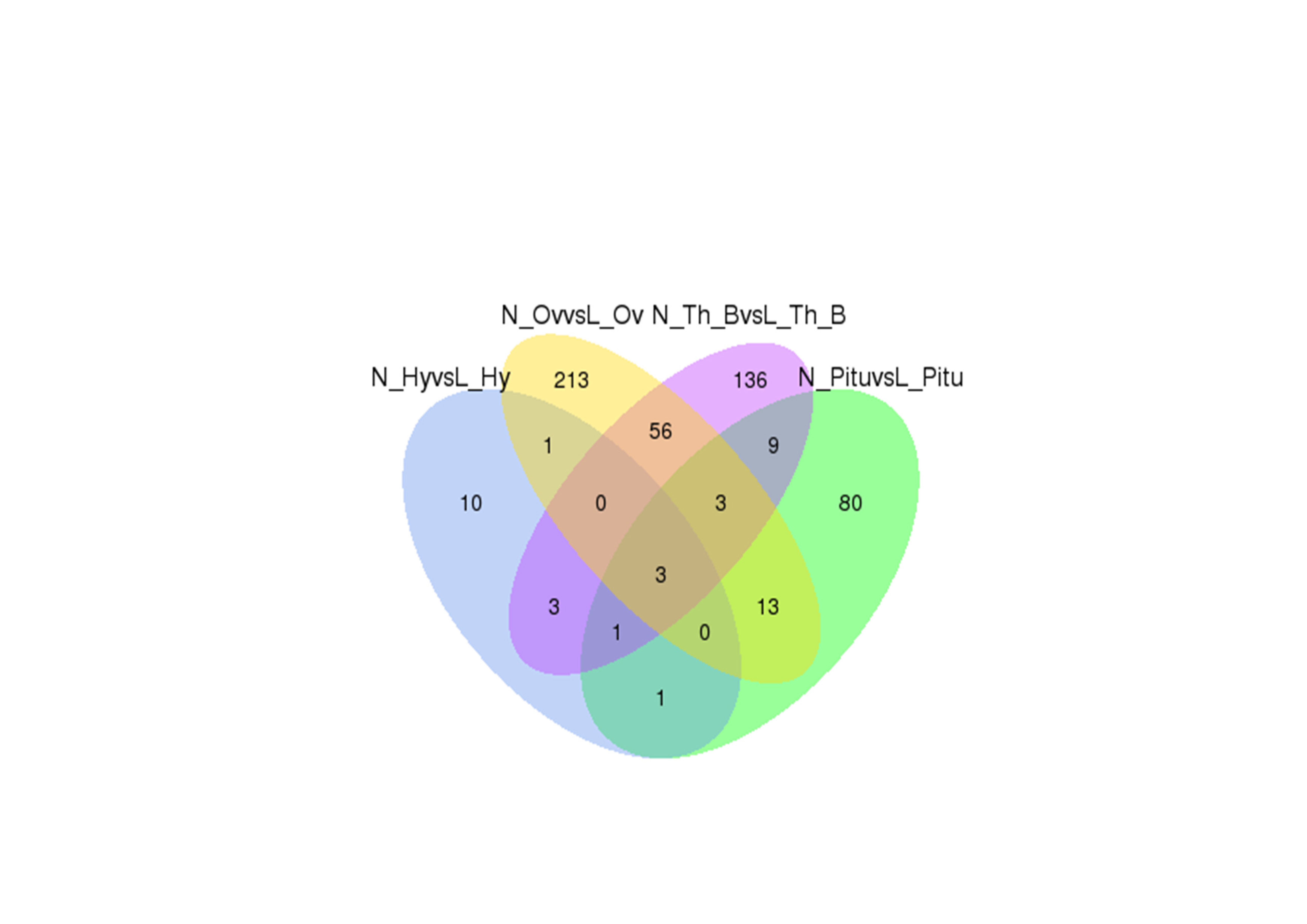

Supplement: S4 Fig — The numbers in each large circle represent the total number of differentially expressed genes (DEGs) for each tissue within the HPGA, and the numbers in the overlapping portions of the circles represent genes that are shared between groups. (TIF) [file pone.0191213.s004.tif]

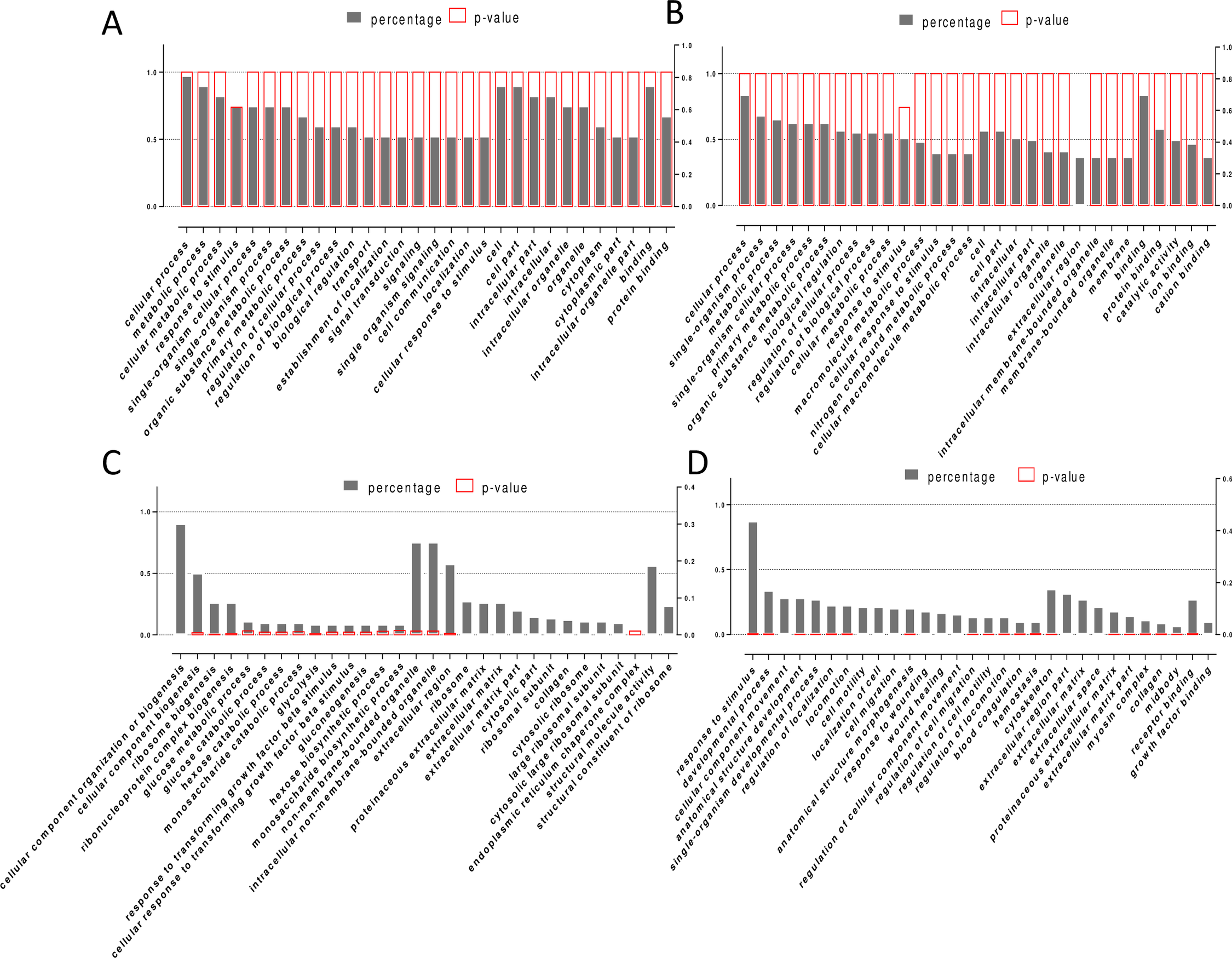

Supplement: S5 Fig — A-D, GO terms distributed in the hypothalamus, the pituitary gland, the stroma ovarii and the walls of follicles (diameter range 8–10 mm). The top 30 GO terms for each comparison group are listed. The GO terms were sorted by the ratio of the number of DEGs enriched in the GO term to the total number of annotated genes enriched in this GO term. All GO terms enriched by the DEGs in each HPGA tissue are provided in S7–S10 Tables. (TIF) [file pone.0191213.s005.tif]
