# Supplementary material for: Transcriptome analysis revealed the possible regulatory pathways initiating female geese broodiness within the hypothalamic-pituitary-gonadal axis
Source: PLoS One. 2018 Feb 6;13(2):e0191213. doi: 10.1371/journal.pone.0191213 (PMC5800542; doi:10.1371/journal.pone.0191213)
Supplement: S1 Table — (DOCX) [file pone.0191213.s006.docx]

**Table S1 Primers used for qRT-PCR**

| **Gene name** |  | **Sequence information** | **Product**  **Length (bp)** | **Annealing temperature (℃)** |
| --- | --- | --- | --- | --- |
| follistatin precursor | F | CTGCAAAGAAACGTGCGAGAA | 141 | 59 |
|  | R | TTCCCATCTAAGCCACACACG |  |  |
| INHBB | F | TGCGTGCAAGAATACGTCGT | 129 | 63.3 |
|  | R | AATGCCTGCCAGTACGAGTGA |  |  |
| zona pellucida C | F | ACGTGAGCAGCAATGCCATC | 159 | 59 |
|  | R | CCTGGATGTTGAGGATGTCGC |  |  |
| asma | F | AGAATAGAGCCTCCAATCCAGACG | 122 | 58.75 |
|  | R | TACCCAGGCATTGCAGACAGG |  |  |
| lipocalin-like | F | GGAAGTGAAGGTGGATAGATACGG | 136 | 58.3 |
|  | R | CCTGCCTCAGACAGACAAGTGC |  |  |
| anti-Mullerian hormone | F | GGTCATCCTGGTGAAGCATTTG | 167 | 58 |
|  | R | TGGTCCTTGGAGCCTCGGTA |  |  |
| inhibin beta B chain precursor | F | TTGGATGTTCAATGTGAGGGC | 120 | 56.3 |
|  | R | GTGTTTGTTATCGGCGAGGC |  |  |
| KPNA2 | F | CTGGTTTGATTCCGAAGTTTGTC | 120 | 54.6 |
|  | R | CTGCCTTTGTTTGTTCTGATGTG |  |  |
| MMP1 | F | CCAACTTATGCCTACACGGACC | 173 | 57.6 |
|  | R | TCTCCACGCAAGGTAGTAATAGCA |  |  |
| hsd3-b | F | GATATGTCTGGCTTTCTGTGGGA | 157 | 56.3 |
|  | R | TCAAGGTCATCTGGATAAGTGTCTG |  |  |
| COL4A2 | F | GGAGGACAGTCGCTGGTTTCT | 168 | 59.4 |
|  | R | TGTATCTGCTGAGGGCTTGCTT |  |  |
| ITGA8 | F | GGAACCCAAAGATGAACCTACAA | 108 | 50 |
|  | R | TGGAGAACTGTGTTGTTGATGGC |  |  |
| osteoglycin | F | TTGTTGGTCTTACACTGCGTTCG | 140 | 53.9 |
|  | R | AGATGTTACCGTGGGAAGCCTG |  |  |
| SNX5 | F | CTATGACGAAAGAGGAGTTTGCCA | 128 | 53.9 |
|  | R | GCTGAGCACAGGGTGAGAAGAA |  |  |
| TMEM2 | R | ACATACAAAGCCACTTGACATCTGC | 121 | 58.3 |
|  | F | CGCCTACTCACAACTGCTCCTG |  |  |
| TMEM132C | F | CTTCCCTGCTCAAGTAGACCTCC | 105 | 56.3 |
|  | R | GCATACATCCCGATCTCCAAAT |  |  |
| TTR | F | GGAACATACAGGGTTGAGTTTGACA | 125 | 50 |
|  | R | GCAATGGTATAATGGCGGTGA |  |  |
| vasopressin V1a receptor | F | TGCCGTAACCTTCCCGAGTG | 144 | 60 |
|  | R | CCAACAACAGGAGCCCAACC |  |  |
| ALDH1A2 | F | GAAAGCCAGCCTCCTTGATGA | 179 | 57 |
|  | R | AAGACATGAACCCATCGGAGTG |  |  |
| PROM1 | F | GACACCATAGTTGGCTACTTCGAG | 163 | 56 |
|  | R | AACCAAACCAGAAGGTATTCACAGA |  |  |
| SFRP2 | F | TCATTCATCTCATCGCAGGTACA | 130 | 56 |
|  | R | CCGAAACAAAGAGCAAGACCA |  |  |
| THBS4 | F | ACGGTGACAGACGATGACTACGC | 132 | 61 |
|  | R | AGGCTCTGCCACAGCTCTGAA |  |  |
| 3β-HSD | F | GACCTGGGGTTTGGAATTGAG | 169 | 60 |
|  | R | TAGGAGAAGGTGAATGGGGTGT |  |  |
| Prolactin(PRL) | F | CCTTGGGGAACTTTTTGACC | 114 | 60 |
|  | R | GTAATGAAACCCCGACCCTG |  |  |
| aquaporin-4-like | F | ATGGGCTGTAATAGACTCTTTGGC | 158 | 60 |
|  | R | CAGTGCTGGTGAGCTGGGATA |  |  |
| B2M | F | TCAGCTCACTTTGCATCTCCATT | 107 | 60 |
|  | R | AGGGCTTGAACACTGAGGCAT |  |  |
| EPB41L1 | F | TCCCTTACCAAATCTTCTGTCCTG | 162 | 60 |
|  | R | GCCATCAACTCTGTCCACTGTCC |  |  |
| npvf | F | CATTGCATCTTCTCCAAGTCTTTC | 114 | 59 |
|  | R | CTGTCAGTCTGTCTCAAGGTGTCC |  |  |
| periplakin | F | TGTTGGTAAGCCAGGCAGTTTC | 130 | 60 |
|  | R | GGTGTTTAGGTATTCGGGTGATG |  |  |

Note: F represents the forward primers, and R represents the reverse primers.
